# Phenytoin-Induced Gingival Overgrowth: A Review of the Molecular, Immune, and Inflammatory Features

**DOI:** 10.5402/2011/497850

**Published:** 2011-07-25

**Authors:** Jôice Dias Corrêa, Celso Martins Queiroz-Junior, José Eustáquio Costa, Antônio Lúcio Teixeira, Tarcilia Aparecida Silva

**Affiliations:** ^1^Department of Oral Surgery and Pathology, School of Dentistry, Federal University of Minas Gerais, 31270-901 Belo Horizonte, MG, Brazil; ^2^Department of Clinical Medicine, School of Medicine, Federal University of Minas Gerais, 31270-901 Belo Horizonte, MG, Brazil

## Abstract

Gingival overgrowth (GO) is a side effect associated with some distinct classes of drugs, such as anticonvulsants, immunosuppressant, and calcium channel blockers. GO is characterized by the accumulation of extracellular matrix in gingival connective tissues, particularly collagenous components, with varying degrees of inflammation. One of the main drugs associated with GO is the antiepileptic phenytoin, which affects gingival tissues by altering extracellular matrix metabolism. Nevertheless, the pathogenesis of such drug-induced GO remains fulfilled by some contradictory findings. This paper aims to present the most relevant studies regarding the molecular, immune, and inflammatory aspects of phenytoin-induced gingival overgrowth.

## 1. Introduction

Gingival overgrowth (GO) comprises any clinical condition in which an increase in the size of the gingiva is observed. Such enlargement can be caused by a multitude of stimuli and stands as a singular complaint in the dental office. Among the drugs that induce GO, the antiepileptic agent phenytoin has been widely related to this condition [1].

Epilepsy is the most common chronic neurological disorder in humans [2]. The prevalence of epilepsy in developed countries reaches approximately 1%, rising to 2% in less developed nations [3]. Epilepsy treatment is based on drug-therapies which aim to help patients to achieve seizure freedom without adverse effects. However, in several cases the first-choice drug fails in the treatment due to a lack of efficacy or to the patient failure to tolerate the medication side effects [4, 5].

The first report of GO associated with the chronic use of phenytoin was made in 1939 [6]. This agent remains as one of the most commonly prescribed medications to treat epilepsy and it may also be used in cases of neuralgias and cardiac arrhythmias [7]. It is estimated that about 30 to 50% of patients taking phenytoin develop significant gingival alterations [8].

Other anticonvulsants have also been associated with GO. Nevertheless, the cases of gingival changes after chronic use of valproic acid, carbamazepine, phenobarbital and vigabatrin in adult patients have been rarely reported [9–11]. Other classes of drugs, such as the immunosuppressant cyclosporine A and some calcium channel blockers (dihydropiridines, diltiazem and verapamil) also have GO as an important adverse effect [12].

To date several studies have tried to determine the pathogenesis of drug-induced GO but the mechanisms that trigger such condition are not fully elucidated. Therefore, the aim of this work was to review the most relevant studies published about phenytoin-induced GO (PGO) and outline the possible mechanisms associated with this condition.

## 2. Clinical and Microscopic Features of the Phenytoin-Induced Gingival Overgrowth

PGO can occur early within 3 months of the drug use and it may reach a state of equilibrium often within the first year of the beginning of medication [13]. PGO seems to be more prevalent in children and teenagers, but there is no difference on its incidence in regard to gender or ethnic groups [14].

PGO incidence and severity is greater in the buccal surface of both upper and lower anterior teeth [15]. Clinically, gingival enlargement begins in the interdental papillae, which increase and coalesce [16]. Tissue appearance may range from a normal aspect to a hyperemic state [17]. Growth is slow but in more severe cases it may go so far as to cover the whole tooth crown [13]. Few cases of PGO have also been reported in edentulous patients [18] and around deciduous teeth [14]. Likewise, there were some reports of GO in areas of dental implants in patients taking phenytoin [19]. 

 Microscopic analysis of PGO biopsies reveals a redundant tissue of apparently regular composition or with an increased amount of collagen and number of fibroblasts. Frequently, the overlying surface epithelium presents rete pegs elongating into the underlying lamina propria [16]. The level of inflammatory cell infiltrate varies significantly [20].

## 3. Correlation between Phenytoin Pharmacological Aspects and Gingival Overgrowth

Many studies correlate the dose of phenytoin with PGO severity [7, 21–23]. Some works suggest a possible positive relationship once the reduction in the prescribed phenytoin dose results in improvement of PGO severity [7]. Other evidence is that patients who present more extensive gingival lesions exhibit higher serum levels of phenytoin when compared to those without PGO [7, 24, 25]. In contrast, some reports showed no correlation between phenytoin daily dose, treatment duration and severity of PGO [26–28]. Similarly, some studies which investigated phenytoin levels in gingival crevicular fluid of epileptic patients were not able to detect any significant difference between PGO and control groups [7, 21]. 

 Given these controversial results, one should look for another explanation for PGO beyond the increased levels of circulating drug. Other theories are based on the hypothesis of a differential tissue response to phenytoin, which would be linked to genetic and environmental factors [29].

## 4. Pathogenesis of PGO

The mechanisms that trigger drug-induced GO have not been completely understood and, although literature data are extensive, they are quite contradictory. 

Some studies demonstrated that drugs such as phenytoin and cyclosporine A are able to inhibit production of extracellular matrix (ECM) by gingival fibroblast and cell proliferation *in vitro* [30, 31]. In contrast, others showed that the accumulation of proteins in ECM, particularly collagen, may occur due to an imbalance between the synthesis and the degradation of ECM, being the possible cause of the GO [32].

**Figure 1:**
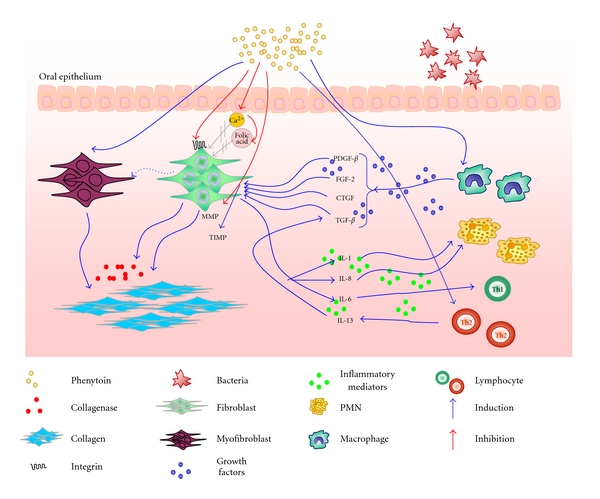
Pathogenesis of phenytoin-induced gingival overgrowth. Several mechanisms are involved in the development of gingival overgrowth. Phenytoin induces a decrease in the Ca^2+^ cell influx leading to a reduction in the uptake of folic acid, thus limiting the production of active collagenase. The drug decreases collagen endocytosis through induction of a lower expression of *α*_2_ *β*_1_-integrin by fibroblasts. Myofibroblasts seem to be stimulated by phenytoin. Other important elements directly responsible for phenytoin-induced gingival overgrowth are cytokines. Phenytoin-activated fibroblasts produce large amounts of IL-6, IL-1, and IL-8. Such mediators are capable of activating the proliferation of T cells and the recruitment of neutrophils to the involved tissues, establishing a direct interaction between the immune system and the connective tissue. This interaction seems to be highly associated with fibrotic diseases. Evidence also points toward a role of dental plaque in the etiology of PGO through induction of a local inflammatory response, which is essential for the gingival overgrowth. Growth factors such as CTGF, PDGF, FGF and TGF-*β* are found in higher levels in fibrotic tissues and play a role in PGO. Phenytoin may affect the production of IL-13 by an activation of Th2 cells, as well as it may induce the release of TGF-*β*, CTGF and other growth factors by macrophages, which leads, synergistically, to fibroblast proliferation, collagen biosynthesis, activation of TIMPs, inhibition of MMPs and ECM synthesis, characteristic processes observed in fibrotic lesions. PDGF-*β*: platelet derived growth factor; FGF-2: fibroblast growth factor-2; TGF-*β*: transforming growth factor-*β*; CTGF: connective tissue growth factor; MMP: matrix metalloproteinase; TIMP: tissue inhibitor of metalloproteinase.

There is some evidence of decreased collagen production after phenytoin administration *in vitro* [33]. Likewise, Kato et al. [34] showed a reduction in mRNA expression of collagen type I and III associated with a higher density of these fibers in the PGO. These results suggest that the imbalance that leads to PGO might be related to decreased collagen degradation, not to an increase on its synthesis (Figure 1).

Collagen fibers are degraded by two pathways: the extracelular one, which occurs by secretion of collagenases; and the intracellular one, by collagen phagocytosis by fibroblasts [35]. In this regard, a common property of the 3 main classes of drugs that induce GO—antiepileptic agents, immunosuppressants and calcium channel blockers—is that they affect calcium metabolism. These drugs induce a decrease in the Ca^2+^ cell influx (due to changes in the sodium-calcium exchange) leading to a reduction in the uptake of folic acid, thus limiting the production of active collagenase [36] (Figure 1). In support to this hypothesis, a study showed that the mRNA collagenase levels are diminished in drug-induced overgrown tissues of rats, with less collagen degradation [37]. Nevertheless, there is a controversy whether therapy with folic acid would have a therapeutic effect on PGO. Inoue and Harrison [38] suggested that patients taking phenytoin and a supplement of folic acid had GO prevented or eliminated. Some other studies showed that patients receiving folate had lower recurrence of gingival enlargement after its surgical removal [39, 40]. In a recent study, Prasad et al. [41] evaluated 60 patients and also concluded that therapy with folic acid is able to delay and minimize GO. In opposition to these data, Brown et al. [42] reported that folic acid was not efficient as a sole therapeutic agent in the reduction of PGO. Similarly, Majola et al. [22] found that serum folate levels did not have any significant association with PGO. These contradictory findings are not sufficient to indicate folic acid therapy as a reliable alternative in the treatment of PGO. 

In the extracellular collagen degradation pathway, other matrix metalloproteinases (MMPs) than collagenase are also responsible for the digestion of collagen fibers [16]. The enzymatic activities of MMPs are controlled by a tissue inhibitor (TIMP) whose function is to antagonize the actions of MMPs [34]. In 2005, Kato et al. [34] showed that the gene expression of MMP-1, 2, and 3 was reduced by phenytoin administration, while the TIMP-1 mRNA was markedly augmented. In accordance, macrophages pretreated with phenytoin and then exposed to LPS had lower production of MMPs than not treated controls [43]. Therefore, this reduction of MMPs is believed to influence the PGO development. 

Regarding the intracellular pathway of collagen degradation, phenytoin significantly decreased collagen endocytosis, which is related to a lower expression of *α*_2_ *β*_1_-integrin [34]. Alpha-2-Beta-1-integrin functions as a specific receptor for collagen type I in fibroblasts and acts in the initial step of collagen phagocytosis, providing an adhesive interaction between fibroblasts and collagen [35]. In this regard, Kataoka et al. [37] also demonstrated that integrin expression is diminished in fibroblasts derived from GO tissues of rats treated with cyclosporine, once more suggesting a role for this pathway in GO (Figure 1). More recently, besides integrins, the receptor uPARAP/ENDO180 was also described as one of the main receptors responsible for collagen phagocytosis [44]. Nevertheless, there is no experimental evidence indicating that this receptor is affected by drugs such as phenytoin.

The possible role of phenytoin on fibroblasts growth and death has also been investigated [45]. During healing, the transition from a granulation tissue to a remodeling tissue requires the apoptosis of fibroblasts. In wounds with inadequate apoptosis the formation of fibrotic tissues may occur. Thus, modulation of apoptosis could contribute to fibrosis in gingival tissues. The study of Kantarci et al. [45] demonstrated that fibroblast apoptosis is decreased in GO, and that this decrease may contribute to fibrosis, particularly in PGO. Thus, increased number of fibroblasts and ECM accumulation appears to be due, in part, to diminished fibroblast death in these tissues [46].

In conclusion, pathogenesis of PGO involves a decrease in collagen degradation which is related to alterations in calcium metabolism, levels of MMPs and TIMPs, integrins expression and fibroblast apoptosis.

## 5. Role of Inflammation and Growth Factors in PGO

An interesting aspect of PGO is that tissues become more fibrotic and have lower levels of inflammation [29]. Therefore, the nature of such overgrowth is close to a fibrosis process that can occur in various organs such as liver, lung and skin [46]. So it is possible to evaluate some features of fibrotic lesions to better understand PGO.

Fibrosis usually results from chronic inflammation—in which inflammation, tissue remodeling and repair processes occur simultaneously [46]. Usually there is a persistent irritant agent that can be infectious, chemical, physical or mechanical. The repair process involves two stages: a regenerative phase and a phase known as fibrosis, in which connective tissues replace normal parenchyma. This phase can become harmful if not properly controlled, resulting in an excessive deposition of ECM [47]. 

Gingival tissues remain frequently in a state of injury and repair, which involves repetitive cycles of production of chemotactic factors, recruitment of inflammatory cells and remodeling. Tissue repair and remodeling are regulated by cytokines and chemokines produced by inflammatory cells such as macrophages and lymphocytes and to a lesser extent by fibroblasts [29]. Based on these findings, many studies have focused on the role of cytokines and growth factors in the pathogenesis of drug-induced GO. It has been shown that phenytoin, nifedipine, and cyclosporine may regulate the expression of cytokines in gingival tissues [48].

Some cytokines and growth factors were found in higher levels in gingival overgrown tissues, including interleukin-6 (IL-6), IL-1, platelet derived growth factor-*β* (PDGF-*β*), fibroblast growth factor-2 (FGF-2), transforming growth factor-*β* (TGF-*β*) and connective tissue growth factor (CTGF) [49–51] (Figure 1). 

 TGF-*β* is a cytokine secreted by several cell types, including macrophages, and with an important role in regulating the collagen metabolism in the connective tissues by stimulating collagen biosynthesis [52]. TGF-*β* is stored within the cell as a homodimer, noncovalently bound to a protein called latency-associated protein (LAP), which maintains TGF-*β* inactive. The dissociation of TGF-*β* and LAP is catalyzed by several agents, such as cathepsins and MMPs. IL-13 induces the formation of latent TGF-*β* and also the production of both cathepsins and MMPs that cleave LAP and activate TGF-*β* [47]. However the effects of TGF-*β* on gingival tissues are surprisingly low if compared with other connective tissues [53]. The hypothesis to explain such finding is that the effects of TGF-*β* in the metabolism of ECM are mediated by CTGF, which is found at high levels in several fibrotic diseases [29]. It was already demonstrated that CTGF levels are increased in PGO tissues [51] and CTGF was also shown to stimulate fibroblast proliferation and ECM synthesis. As demonstrated, there is a requirement to simultaneously use CTGF and TGF-*β* in skin to induce fibrosis [54]. Thus, neither of the two factors alone was able to initiate the fibrotic processes. CTGF is rapidly and potently induced by TGF-*β* and contributes to the regulation of some genes in the ECM [55]. Although CTGF is continuously present in the tissue, TGF-*β* is only found in the initial periods, indicating that there may be a cascade effect [51]. The mechanism by which CTGF promotes fibrosis has been investigated and a possible interaction with integrins has also been proposed [56] (Figure 1).

A recent study shows a possible association between GO and epithelial mesenchymal transition (EMT) [57]. EMT is a process in which epithelial cells *trans-*differentiate into fibroblast-like cells. TGF-*β*1 is a potent inducer of EMT in a variety of tissues and CTGF expression is increased in cells undergoing EMT [57]. Then, the observation of elevated levels of EMT markers in the PGO lesions could suggest the EMT contribution in human GO and fibrosis [57].

Experimental studies in animals also have demonstrated a role for Th2-immune responses and cytokines IL-4, IL-13, IL-5, and IL-21 in fibrotic processes [47]. Interestingly, phenytoin has complex effects on the immune system and it was already observed an induction of Th2 response by this drug. Phenytoin-treated mice were shown to have increased production of IL-4 and decreased levels of IL-1*α* and Interferon-*γ* (IFN-*γ*) [58].

Phenytoin also increases the production of IL-6 and IL-8 by fibroblasts [8]. IL-6 is capable of activating the proliferation of T and B cells, and it has been associated with fibrosis in various organs. The increase of IL-6 levels seems to involve the cyclooxygenase-2 (COX-2) pathway [59] (Figure 1).

IL-8 is chemotactic for polymorphonuclear neutrophils (PMN) and T cells and it has also been associated with fibrosis in the liver and kidney [60, 61]. The number of PMN and T cells is increased in gingival tissues induced by phenytoin [62], so phenytoin could contribute to the increased recruitment and activation of these cells by upregulation of IL-6 and IL-8 [8].

Besides cytokines, other important proteins involved in PGO development are cathepsins and lysosomal proteases, responsible for digestion of 90% of cellular proteins. A rare congenital disease, mucolipidoses II, characterized by impairment on cathepsin maturation and, thus, by reduction of its activity, has the GO as one of its features [63]. An *in vitro* study showed that phenytoin and cyclosporine can inhibit the production of cathepsin L, but not B, by gingival fibroblasts [63] and transgenic mice deficient for cathepsin L spontaneously develop GO [64].

Another interesting element linking systemic fibrotic diseases to GO is the presence of myofibroblasts in phenytoin-induced tissues [65]. Myofibroblasts are highly differentiated cells that secrete large quantities of collagen and are involved in the tissue repair process [47]. The presence and role of myofibroblasts in other fibrotic diseases, such as skin fibrosis, were already known [47]. The presence of myofibroblasts in PGO suggests that this drug exacerbates the normal tissue turnover/wound healing signals responsible for the appearance of myofibroblasts [65].

Based on these findings, it is possible to conclude that the process that leads to PGO involves a fine regulation of growth factors and inflammatory cytokines, as shown for other fibrotic diseases. Understanding the relationship of the immune system with the fibrotic process and how phenytoin affects it seems to be a crucial step to control this phenomenon.

## 6. Bacterial Biofilm and PGO Development

The role of dental plaque as a co-factor in the etiology of PGO was recognized in the latest periodontal disease classification system [66]. In such classification system, the “drug-induced gingival diseases” were categorized into the major group “dental plaque-induced gingival diseases” and their development and severity were pointed as being influenced by the accumulation of dental plaque [66].

Despite this classification system, the literature is still controversial whether the oral biofilm is important to trigger or to worsen GO. Some studies have found significant correlations between the incidence and/or severity of GO and the amount of accumulated dental plaque and calculus [67, 68]. Other studies have shown that satisfactory oral hygiene is able to reduce the overgrowth, but not to completely prevent it [69, 70]. A possible involvement of some bacteria species in the gingival lesion development was previously studied. Takada et al. [71] found that *Prevotella intermedia* (Pi) prevalence is higher in patients with PGO than in patients taking the medication but who did not develop that injury, and also those who did not use phenytoin. Akiyama et al. [72] also examined subgingival bacterial profiles in subjects taking phenytoin who exhibited, or not, PGO. Quantitative analysis performed before and after periodontal treatment, revealed two bacterial species significantly associated with PGO, *Treponema denticola* (Td) and *Porphyromonas gingivalis* (Pg). Miyazaki also found increased levels of Td and Pg in severely affected PGO sites [73]. These findings are in contrast to the studies of Yamada et al. [24] and Smith et al. [74] who did not find differences in the oral microbial population of patients with and without PGO. By examining these conflicting data it does not seem reasonable to assume that there is a specific group or specific bacteria that lead to GO. More studies are warranted in this field in order to clarify the role of bacteria on PGO. 

 In addition to bacteria itself the host response to these antigens also may be related to PGO and has received increasing attention. The bacterial components in dental plaque can be recognized by host cell Toll-like receptors (TLRs), which are sensors of pathogen-associated molecular patterns [75]. In this regard, phenytoin demonstrated some modulating effects over the TLRs. An *in vitro* study using hamster cells treated with phenytoin and cyclosporine showed opposite results for these two substances. While cyclosporine increased signaling by TLR2 and TLR4, phenytoin decreased this signaling with lower expression of adhesion molecules such as CD54 [75]. Recognition of the cell-wall components peptidoglycan and lipoproteins by TLR2 and recognition of the outer membrane component lipopolysaccharide (LPS) by TLR4 lead to a series of events, including nuclear factor (NF)-*κ*B activation, that results in cytokine production and expression of adhesion molecules in fibroblasts [75]. The reduction in cell signaling induced by phenytoin may alter the inflammatory response in gingival tissues, favoring bacterial invasion and proliferation and, therefore, may be an important factor in the pathogenesis of PGO. Here, it seems reasonable to conclude that the interaction between dental plaque and the host response to it has a role in the etiology of PGO, but this relationship is not completely elucidated.

## 7. Genetic Factors Associated with PGO

As mentioned before, not all patients taking phenytoin develop PGO. Evidence suggests that genetic factors also might have a significant role in the pathogenesis of PGO and in the patient's susceptibility to this unwanted effect. A genetic predisposition could influence a variety of factors in the drug-plaque-induced inflammation. These include gingival fibroblast functional heterogeneity, collagenolytic activity, drug metabolism and collagen synthesis [17, 76].

One study examined the prevalence and correlation of the polymorphism of integrin genes and drug-induced GO [35]. It has been shown that patients with the genotype C 807 showed lower expression of integrins and those with GO had a higher frequency of the allele 807 C, suggesting that the integrin has a critical role in the induction of drug-induced GO [35].

Regarding drug metabolism, the cytochrome P450 (CIP2C9 and 2C19) is responsible for phenytoin conversion on its hydroxylated form in the liver [25]. Pharmacogenetic influences on drug metabolism have been widely reviewed and gene polymorphism of cytochrome P450 2C19 appeared to be responsible for much of the interindividual variability on drug elimination [77]. Polymorphisms in CIP2C9 were reported and particularly the 2C9*3 polymorphism has been suggested to exert great influence on the metabolism of phenytoin, so it was hypothesized to be a candidate gene for prevention and early diagnosis of the GO severity [78]. Soga et al. [25] showed in a study with 28 patients taking phenytoin that those who had the expression of the polymorphism CIP2C9*3 had higher serum concentrations of phenytoin. However, among patients with this polymorphism only a small proportion exhibited GO. 

 In relation to fibroblasts, since 1981 Hassell [79] stimulated the discussion about possible phenotypic differences between gingival fibroblasts. It has been speculated that drug-induced GO may occur due to direct or indirect stimulation of the proliferation of some populations of fibroblasts, called “responsive”, which somehow justified the individual susceptibility to those drugs [80–82]. Cell culture studies made with monozygous and dizygous twins have confirmed the functional heterogeneity of gingival fibroblasts [82].

## 8. Concluding Remarks

The review of various investigations into the pathogenesis of drug-induced GO supports the hypothesis that it is a side effect with a multifactorial etiology. The inflammatory changes that occur within gingival tissues seem to orchestrate the interaction between drugs and fibroblasts. 

 Further studies are required for a better understanding of the aspects of gingival extracellular matrix metabolism and the interactions between drugs, immune response, cytokines, growth factors and gingival cells. As treatment and prevention of drug-induced GO remains unsatisfactory, for some patients, the change in drug therapy should be considered, a plaque control program might be performed and, in some cases, surgical elimination of the gingival tissue must be unavoidable yet. A thorough understanding of the pathogenesis of this unwanted side effect is essential if we are to devise appropriate regimens for its prevention and treatment.

##  Conflict of Interests 

The authors declare that they have no conflict of interest.
